# Genome-wide identification of resistance genes and transcriptome regulation in yeast to accommodate ammonium toxicity

**DOI:** 10.1186/s12864-022-08742-y

**Published:** 2022-07-15

**Authors:** Wenhao Fu, Xiuling Cao, Tingting An, Huihui Zhao, Jie Zhang, Danqi Li, Xuejiao Jin, Beidong Liu

**Affiliations:** 1 State Key Laboratory of Subtropical Silviculture, College of Forestry and Biotechnology, Zhejiang A&F University, Lin’an, Hangzhou, 311300 China; 2 Department of Chemistry and Molecular Biology, University of Gothenburg, Medicinaregatan 9C, SE-413 90 Goteborg, Sweden; 3 Center for Large-Scale Cell-Based Screening, Faculty of Science, University of Gothenburg, Medicinaregatan 9C, SE-413 90 Goteborg, Sweden

**Keywords:** *Saccharomyces cerevisiae*, Ammonium, NH_4_Cl, Genome-wide screen, Transcriptome

## Abstract

**Background:**

Ammonium is an important raw material for biomolecules and life activities, and the toxicity of ammonium is also an important ecological and agricultural issue. Ammonium toxicity in yeast has only recently been discovered, and information on its mechanism is limited. In recent years, environmental pollution caused by nitrogen-containing wastewater has been increasing. In addition, the use of yeast in bioreactors to produce nitrogen-containing compounds has been developed. Therefore, research on resistance mechanisms that allow yeast to grow under conditions of high concentrations of ammonium has become more and more important.

**Results:**

To further understand the resistance mechanism of yeast to grow under high concentration of ammonium, we used NH_4_Cl to screen a yeast non-essential gene-deletion library. We identified 61 NH_4_Cl-sensitive deletion mutants from approximately 4200 mutants in the library, then 34 of them were confirmed by drop test analysis. Enrichment analysis of these 34 genes showed that biosynthesis metabolism, mitophagy, MAPK signaling, and other pathways may play important roles in NH_4_Cl resistance. Transcriptome analysis under NH_4_Cl stress revealed 451 significantly upregulated genes and 835 significantly downregulated genes. The genes are mainly enriched in: nitrogen compound metabolic process, cell wall, MAPK signaling pathway, mitophagy, and glycine, serine and threonine metabolism.

**Conclusions:**

Our results present a broad view of biological pathways involved in the response to NH_4_Cl stress, and thereby advance our understanding of the resistance genes and cellular transcriptional regulation under high concentration of ammonium.

**Supplementary Information:**

The online version contains supplementary material available at 10.1186/s12864-022-08742-y.

## Background

Ammonium is a building block for amino acids, nucleic acids, polysaccharides, and other important cellular structural components in all organisms, and at the same time is also an important raw material for the synthesis of secondary metabolites such as alkaloids and polyamines [1]. Ammonium is a paradoxical nutrient ion. First, ammonium is the key nitrogen source for all life forms, including bacteria, fungi, protists, plants, and animals. Whereas the ammonium used by plants mainly comes from natural nitrogen fixation, in heterotrophic cells, it is mainly derived from cellular metabolites [2, 3]. Second, high concentrations of ammonium are toxic to organisms [4–6]. The downstream molecular events of NH_4_^+^ as a nutrient component have been extensively studied; it is mainly related to biosynthesis, carbon metabolism, energy metabolism, primary nitrogen metabolism, plant hormones, cell wall stability, and signaling pathways [7]. However, the mechanism underlying ammonium toxicity, and how to inhibit it, are areas of intense current research [8–10].

In plants, excessive NH_4_^+^ can inhibit photosynthesis, block the growth of plant roots and leaves [11], and promote the accumulation of reactive oxygen species [12]. When ammonium is the primary nitrogen source, it usually significantly inhibits plant growth, manifesting as a phenotype of severely inhibited root growth and chlorosis, which is a well-known manifestation of ammonium salt toxicity [13]. For vertebrates, high concentrations of ammonium may displace K^+^ and depolarize neurons, leading to the activation of NMDA-type glutamate receptor, which leads to excessive Ca^2+^ influx and subsequent cell death in the central nervous system, ultimately leading to convulsions, coma, and even death [6]. In addition, ammonium salts are also important environmental pollutants in the ecosystem. Excessive use of ammonium fertilizer will not only cause soil acidification, resulting in the loss of soil nutrients and the aggravation of the harm of heavy metal pollution, but also lead to secondary salinization of the soil, resulting in large-scale reductions in crop yield and deterioration of quality [14].

The harm of ammonium to animals and plants has long been known, and extensively studied; however, the discovery of ammonium toxicity in *Saccharomyces cerevisiae* is relatively recent [15]. The reason for this is that yeast cells have extremely high tolerance to ammonium under normal culture conditions [16]. In *Saccharomyces cerevisiae*, ammonium uptake is facilitated by ammonium permeases (Mep1, Mep2, and Mep3), which transport NH_4_^+^ ions and conduct ATP-dependent export of protons to maintain intracellular charge balance and pH. This process can put a great burden on the energy metabolism of cells, especially when there is an excess of ammonium salts outside the cells [17]. With growing interest in the biosynthesis of nitrogen-containing compounds using yeast bioreactors, the mechanisms of ammonia resistance in yeast cells have also received increased attention [18]. Therefore, it is particularly important to fully understand the mechanism of action of ammonium metabolism in yeast cells and to find and discover resistance genes and pathways. This could aid in the development of ways to increase cellular ammonia resistance while reducing intracellular energy expenditure.

To gain a comprehensive and in-depth understanding of the causes and mechanisms underlying ammonium toxicity in yeast cells, we performed experiments using two genome-wide strategies. First, we used the yeast gene deletion library (SGA collection) to screen NH_4_Cl resistance-related mutants. Next, we analyzed transcriptome changes in yeast cells at concentrations of ammonium that significantly inhibited growth. Finally, the present study reveals the key genes and cellular pathways underlying resistance to NH_4_Cl in the yeast genome, which provides an experimental basis for further cultivating high ammonium resistant strains and designing schemes to reduce ammonium toxicity.

## Results

### Genome-wide screen for NH_4_Cl-sensitive mutants

**Fig. 1:**
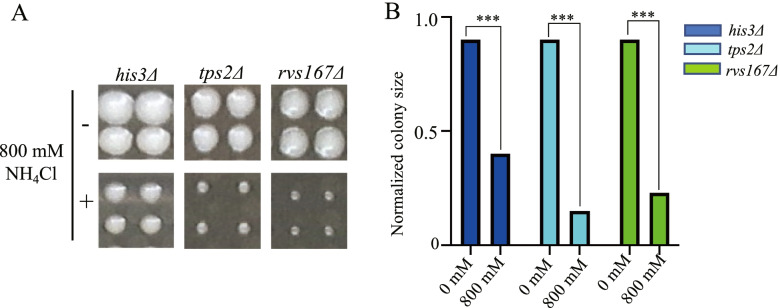
Images of colony growth on plates with or without 800 mM NH_4_Cl. **A** Phenotypes of SGA control strain and the mutants in our screen, and each mutant has four adjacent clones. **B** The cell area of *his3Δ, tps2Δ,* and *rvs167Δ* under NH_4_Cl showed reductions of 52, 75 and 67%, respectively, compared with the control

To understand the specific mechanism of yeast under NH_4_Cl stress, we used the SGA-V2 library to screen for mutants that are sensitive to NH_4_Cl. First, we diluted the control strain *his∆* in plates with a gradient of concentrations of NH_4_Cl. The growth of the strain began to be inhibited under a concentration of 500 mM NH_4_Cl (Fig. S1). This is consistent with previous reports that yeast have high NH_4_Cl tolerance [15]. After determining the concentration range of NH_4_Cl inhibition on yeast, one plate (SGA-V2–3) from the library was randomly selected for a preliminary experiment. The NH_4_Cl concentration was chosen with reference to our previous criteria [19]; that is, the colony size of the control strain was reduced by nearly half, and some mutants showed significant growth inhibition. This criterion was met when the NH_4_Cl concentration reached 800 mM (Fig. 1), so we chose 800 mM NH_4_Cl as the treatment concentration for the genome-wide screen. About 4200 genes in the yeast gene deletion collection were screened. Based on the criteria described in the method, a total of 61 mutants were considered sensitive (Table S1).

### Drop test verifies high-throughput screening results

**Fig. 2:**
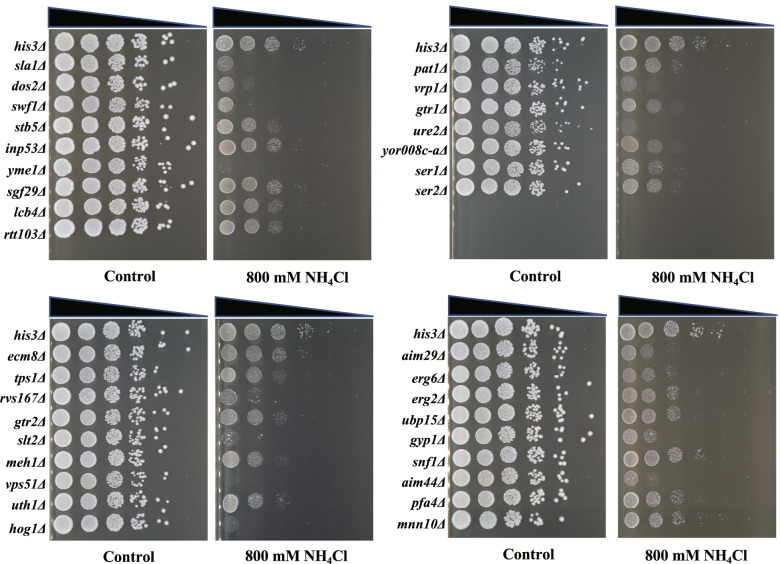
Spot test to verify screening results. Cells were started from OD_600_ = 0.5 and then serially diluted ten times. The 5 μl diluent was spotted onto YPD containing 800 mM NH_4_Cl. Plates were photographed after 48 h incubation at 30 °C

To verify the reliability of the high-throughput screening results, we performed drop test experiments on each of the 61 deletion strains. Among these deletion strains, 34 remained sensitive to NH_4_Cl in the drop test experiments (Fig. 2). Growth of these 34 deletion strains was inhibited, compared with control strains, in the presence of 800 mM NH_4_Cl. Information on these 34 mutants, together with their quantitative fitness scores in genome-wide screening, are summarized in Table 1. Based on the results of the drop test, we classified the degree of NH_4_Cl inhibition of the mutants. Colony growth only in the first column was defined as “serious” inhibition, colony growth only in the first two columns was defined as “obvious” inhibition, “moderate” referred to colony growth in the first three columns, and “slight” referred to the first four columns with colony growth. Seven of the strains showed a relatively serious inhibitory effect, including *sla1Δ*, *swf1Δ*, *yme1Δ*, *slt2Δ*, *hog1Δ*, *vps51Δ,* and *ure2Δ*. In the other 27 mutants, 10 strains were obviously inhibited (*dos2Δ*, *rvs167Δ*, *vrp1Δ*, *gtr1Δ, yor008c-aΔ, ser1Δ*, *ser2Δ*, *aim29Δ*, *gyp1Δ,* and *aim44Δ*); 11 strains were moderately inhibited (*stb5Δ*, *tps1Δ*, *gtr2Δ*, *meh1Δ*, *uth1Δ*, *pat1Δ*, *erg6Δ*, *erg2Δ*, *ubp15Δ*, *pfa4Δ,* and *mnn10Δ*); and 6 strains were slightly inhibited (*inp53Δ*, *sgf29Δ*, *lcb4Δ*, *rtt103Δ*, *ecm8Δ*, and *snf1Δ*) (Fig. 2). The deleted genes in the various mutants were distributed in a variety of different cellular pathways, indicating that cellular mechanisms involved in NH_4_Cl cellular resistance involve not single, but multiple, pathways.

**Table 1:** Results for the 34 NH_4_Cl-sensitive mutants in genome-wide screening

ORF	Gene	Score 1	***p***-Value 1	Score 2	p-Value 2	Score 3	***p***-Value 3
YBR076W	ECM8	− 0.7769	0.00026	− 0.3286	0.00001	− 0.4883	0.00002
YBR126C	TPS1	−0.7275	0.00308	−0.3579	0.00003	−0.3630	0.00002
YBL007C	SLA1	−0.5340	0.00002	−0.2500	0.00003	−0.4729	0.00042
YCL010C	SGF29	−0.3755	0	−0.2031	0.00001	−0.2475	0.00011
YCR077C	PAT1	−0.3581	0.00001	−0.3037	0.00001	−0.4301	0.00004
YDR245W	MNN10	−0.5099	0.00003	−0.2407	0.0001	−0.3961	0.00001
YDR289C	RTT103	−0.5253	0.0002	−0.3517	0	−0.2833	0.00002
YDR388W	RVS167	−0.9690	0.00025	−0.6730	0	−0.5855	0.00003
YDR068W	DOS2	−0.6344	0.00041	−0.2702	0.00005	−0.3192	0.00006
YDR126W	SWF1	−0.6447	0.00021	−0.3695	0	−0.2695	0
YGR163W	GTR2	−0.4364	0.0001	−0.3281	0.00016	−0.6712	0.0002
YGR208W	SER2	−0.4953	0.0002	−0.4509	0.0001	−0.5924	0.00009
YHR030C	SLT2	−0.8645	0.00024	−0.6444	0.00003	−1.2245	0.00167
YHR178W	STB5	−0.2197	0	−0.2309	0.00008	−0.4497	0.00017
YKR007W	MEH1	−0.4576	0.00001	−0.3163	0.00001	−0.5692	0.00006
YKR020W	VPS51	−0.4114	0.00002	−0.5171	0.00008	−0.4268	0.00019
YKR042W	UTH1	− 0.3739	0	− 0.5003	0.00002	−0.5051	0.00001
YKR074W	AIM29	−0.3547	0	−0.2324	0	−0.3205	0.00001
YLR113W	HOG1	−0.6609	0.00002	−0.7480	0.00001	−0.7253	0
YLR337C	VRP1	−0.4252	0.00001	−0.4744	0.00007	−0.5412	0.00001
YML008C	ERG6	−0.2919	0.00004	−0.3039	0.00008	−0.3965	0.00037
YML121W	GTR1	−0.3969	0.00002	−0.5714	0.00012	−0.6559	0.00006
YMR202W	ERG2	− 0.2826	0.00002	−0.3973	0.00001	− 0.3263	0.00004
YMR304W	UBP15	−0.3160	0.00001	−0.2987	0.00022	−0.3343	0.00004
YNL229C	URE2	−0.3338	0	−0.3416	0	−0.5365	0.00001
YOL003C	PFA4	−0.2668	0	−0.2376	0.00008	−0.2846	0.00005
YOR008C-A	–	−0.3447	0.00001	−0.3527	0.00013	−0.4377	0.00005
YOR070C	GYP1	−0.4937	0.00069	−0.5041	0.00006	−0.2866	0.00009
YOR109W	INP53	−0.2873	0.00001	− 0.3000	0.00003	−0.2175	0.00009
YOR171C	LCB4	−0.2718	0.00008	− 0.3479	0.00002	−0.3126	0.00005
YOR184W	SER1	−0.4764	0.00009	−0.4294	0.00002	−0.3150	0.00008
YPR024W	YME1	−0.2942	0.00037	−0.2608	0.00203	−0.3263	0.00017
YDR477W	SNF1	−0.2626	0.00009	−0.3186	0.00001	−0.2766	0.00005
YPL158C	AIM44	−0.2431	0.00001	−0.2696	0.00003	−0.4340	0.00015

**Fig. 3:**
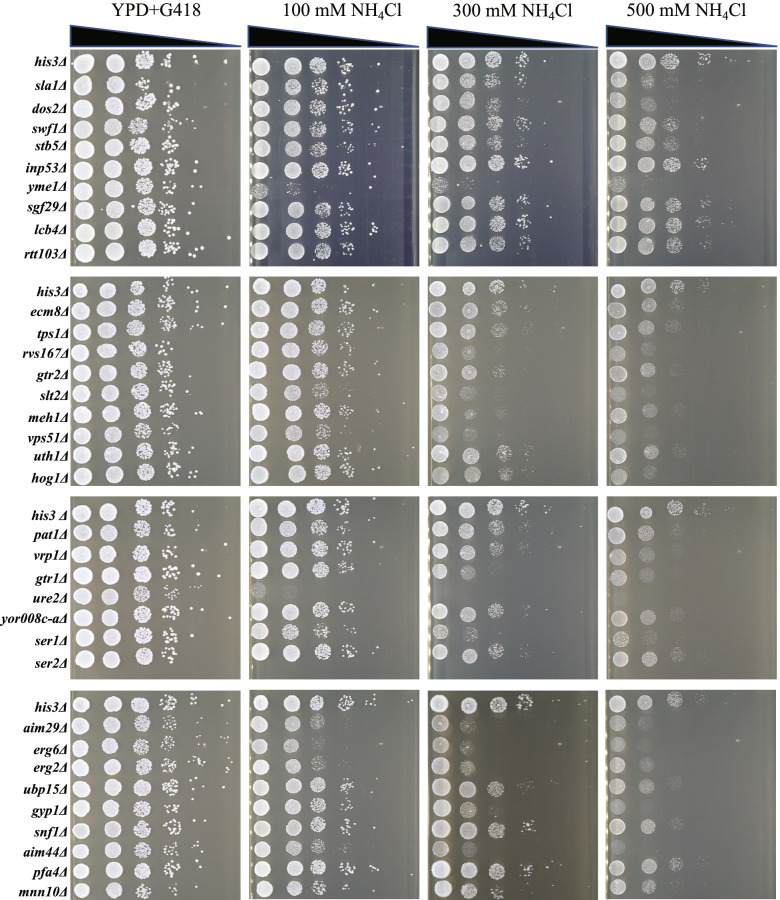
Spot test of the 34 selected deletion mutants under different concentrations of NH_4_Cl. Each strain was serially diluted in a 10-fold gradient and 5 μl were spotted onto differently treated agar plates. Plates were incubated at 30 °C and photographed after 48 h

Since low concentrations of NH_4_Cl have no obvious inhibitory effect on yeast growth, we used a high concentration of 800 mM NH_4_Cl for high-throughput screening. This concentration resulted in an obvious inhibitory effect on wild-type yeast. To verify the sensitivity of the mutants we screened to NH_4_Cl, we performed gradient NH_4_Cl experiments on the 34 mutants. A concentration gradient of 0, 100, 300, and 500 mM NH_4_Cl was set up for the drop test, in accordance with the previous serial dilution method. At a concentration of 100 mM, the growth of *yme1Δ* and *ure2Δ* was severely inhibited, indicating that these two genes are essential for cellular resistance to NH_4_Cl toxicity (Fig. 3). In addition, *rvs167Δ*, *slt2Δ*, *vps51Δ*, *gtr1Δ*, *ser1Δ*, *aim29Δ*, *erg2Δ*, *erg6Δ*, *gyp1Δ,* and *aim44Δ* were significantly inhibited at a concentration of 300 mM NH_4_Cl. At a concentration of 500 mM NH_4_Cl, most of the 34 mutants showed growth inhibition, while the growth of the control strain was still relatively normal, indicating that the 34 genes obtained by high-throughput screening played different roles in the resistance of cells to NH_4_Cl. Deletion of these genes makes cells sensitive to different degrees of NH_4_Cl stress. Previous studies have revealed a set of multidrug resistant genes whose deletions are associated with sensitivity to multiple compounds with diverse modes of action that are utilized by cells in a wide range of stress responses [20–22]. Among the 34 genes corresponding to sensitive deletion mutants, 13 were previously thought to contribute to general multidrug resistance, including *ERG2*, *ERG6*, *GTR1*, *PAT1*, *RVS167*, *SLA1*, *SLT2*, *SNF1*, *URE2*, *VPS51*, *VRP1*, *YKR007W*, and *YME1*.

### Classification of genes related to NH_4_Cl resistance

**Fig. 4:**
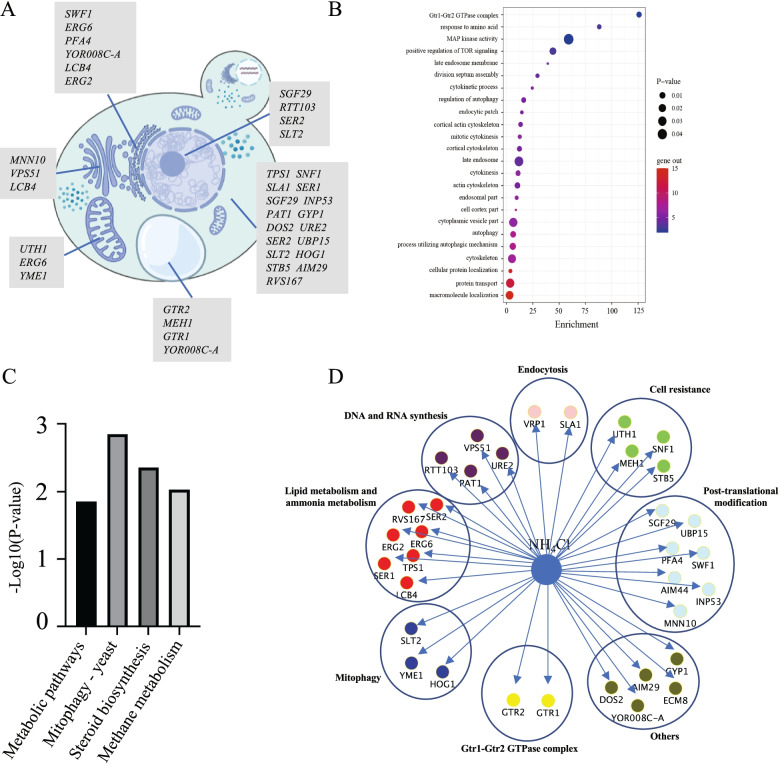
Bioinformatic analysis of 34 NH_4_Cl-sensitive mutant related genes. **A** Gene mapping of the 34 genes in the yeast cell. These genes are distributed in the cytoplasm, nucleus, Golgi, vacuole, endoplasmic reticulum, and mitochondrion. **B** Bubble plot of GO term analysis for the 34 genes. The horizontal axis marks the fold enrichment of the screened genes in the corresponding GO terms. The redder the color, the more genes are enriched in the GO term. Smaller bubbles represent lower *P*-values. **C** KEGG analysis for the 34 genes [23]. **D** Functional annotation for the 34 genes

To further understand the function of the screened genes, we conducted a bioinformatics analysis of these genes. First, to understand the localization of these genes, we analyzed and mapped all of the genes for the cellular locations of their encoded proteins (Fig. 4A). Most of the gene products were located in the cytoplasm, nucleus, and endoplasmic reticulum (ER). In addition, *MNN10* and *VPS51* were located in the Golgi apparatus; *GTR1*, *GTR2,* and *MEH1* were located in the vacuole; and *UTH1* and *YME1* were located in the mitochondria. Further, several of the identified genes encoded products localized to more than one cellular location: *SGF29*, *SER2,* and *SLT2* were distributed in both the cytoplasm and the nucleus; *YOR008C-A* was located in both the ER and vacuole; *LCB4* was located in both the ER and Golgi apparatus; and *ERG6* was distributed in both the ER and mitochondria. GO and KEGG analysis of the 34 genes found that they were mainly enriched in categories related to Gtr1-Gtr2 GTPase complex, cytoskeleton, MAPK activity, TOR signaling, mitophagy, and metabolic pathways (Fig. 4B and C, Table S2). In addition, functional classification of the 34 resistance genes was performed according to the functional description of the Saccharomyces genome database (https://www.yeastgenome.org/) (Fig. 4D). The genes for five of the deletion mutants were unidentified in terms of function. Of the remaining 29, the genes could be divided into seven groups: lipid metabolism- and ammonia metabolism-related genes, post-translational modification, mitophagy, Gtr1-Gtr2 GTPase complex, DNA and RNA synthesis-related genes, and cell resistance- and endocytosis-related genes. Overall, this analysis revealed the complexity of the machinery involved in resistance to NH_4_Cl toxicity. NH_4_Cl may influence cells by affecting both growth and metabolism, and the integrity of these pathways plays an important role in cells’ resistance to ammonium toxicity.

### Transcriptome analysis in response to NH_4_Cl stress

In order to further understand the mechanism of NH_4_Cl toxicity on cells, we cultured wild-type strain BY4741 with different concentrations of NH_4_Cl to find suitable processing conditions for transcriptome sequencing (Fig. S2). Under the condition of 800 mM NH_4_Cl treatment, the cells grew for 8 hours and entered the logarithmic growth phase. At this time, the effect of NH_4_Cl on the cells was already reflected at the transcription level. Therefore, transcriptome sequencing analysis was performed using this treatment condition, and untreated samples grown at the same time were used as a control.

**Fig. 5:**
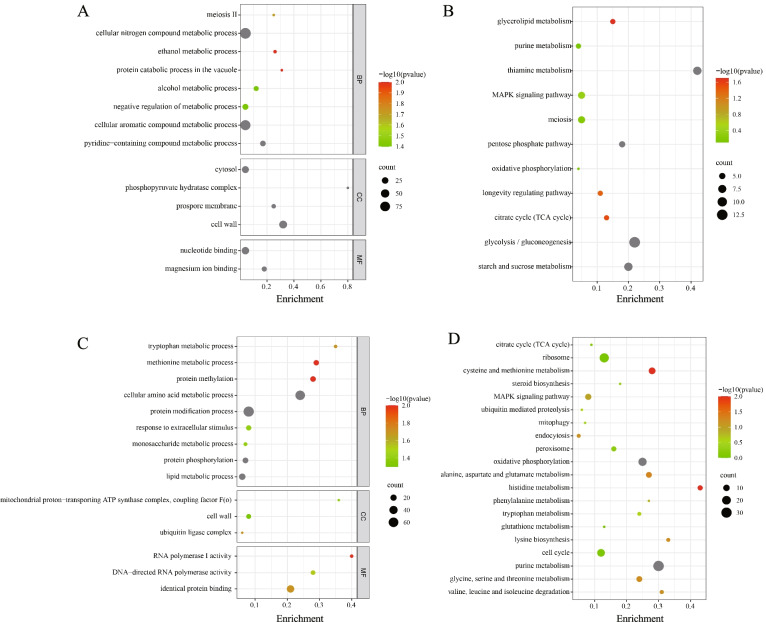
Scatter map of enrichment pathways. **A** GO enrichment analysis of 451 upregulated genes. **B** KEGG enrichment analysis of 451 upregulated genes. **C** GO enrichment analysis of 835 downregulated genes. **D** KEGG enrichment analysis of 835 downregulated genes. BP, CC, and MF represent Biological Process, Cellular Component, and Molecular Function groups of GO, respectively. The grey scatter node represents -log10(*p*-value) > 2

We analyzed the expression levels of 5966 genes in total, of which there were 451 significantly upregulated genes and 835 significantly downregulated genes. Then, we performed GO and KEGG analysis on the 451 significantly upregulated genes. The main enrichment pathways were cellular nitrogen compound metabolic process, cell wall, MAPK signaling pathway, oxidative phosphorylation, and TCA cycle (Fig. 5A and B). GO and KEGG analysis of 835 significantly downregulated genes found that these genes were mainly enriched in ubiquitin-mediated proteolysis, protein phosphorylation, lipid metabolic process, mitophagy, and glycine, serine, and threonine metabolism (Fig. 5C and D). It has been demonstrated that under stress, cells tend to optimize cellular resources for stress adaptation, leading to the massive expression of genes involved in stress adaptation accompanied by inhibition of expression of genes involved in proliferation and cell cycle progression [24].

**Fig. 6:**
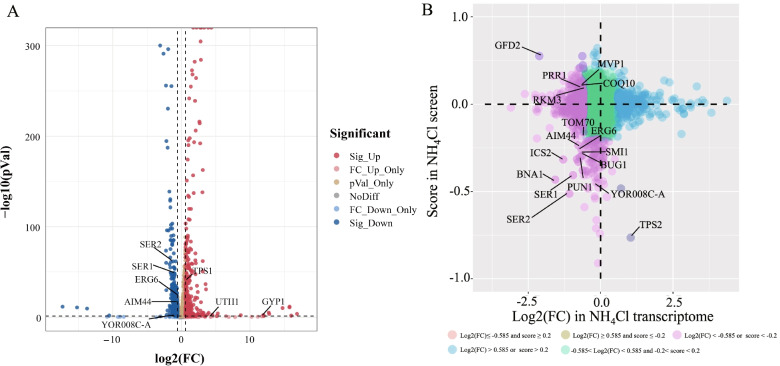
Correlation analysis of high-throughput screening and transcriptome sequencing. **A** Volcano map distribution of differentially expressed genes. **B** Correlation analysis of two studies. The horizontal axis represents LogFC of each gene in our transcriptome analysis experiments. The vertical axis represents mean score of mutants from three replicates in our screen. Different colors represent different groups with certain classification features

To better compare our screening and transcriptome sequencing analysis results, we performed transcriptomic analysis of the expression of the 34 deleted genes. The significantly upregulated genes were *TPS1*, *UTH1,* and *GYP1*. The significantly downregulated genes were *SER1*, *SER2*, *ERG6*, *AIM44,* and *YOR008C-A*. The genes for which expression levels were not significantly downregulated were *ECM8*, *SLA1*, *SGF29*, *PAT1*, *MNN10*, *RTT103*, *RVS167*, *DOS2*, *SWF1*, *GTR2*, *SLT2*, *STB5*, *MEH1*, *VPS51*, *AIM29*, *HOG1*, *VRP1*, *GTR1*, *ERG2*, *UBP15*, *URE2*, *PFA4*, *INP53*, *LCB4*, *YME1,* and *SNF1* (Fig. 6A). Among the 34 genes screened, most showed no obvious expression changes in the transcriptome, a lack of such correlation was also previously reported [25].

Next, a correlation estimate was performed for all genes identified in both the mutant screening and transcriptomic analysis. As shown (Fig. 6B), the genes with significantly upregulated expression and score ≤ − 0.2 were *TPS1* and *TPS2*, and the genes with significantly downregulated expression and score ≤ − 0.2 were *BNA1*, *ICS2*, *AIM44*, *TOM70*, *ERG6*, *BUG1*, *PUN1*, *SMI1*, *YOR008C-A*, *SER1,* and *SER2*. The genes with significantly downregulated expression and score ≥ 0.2 were *GFD2*, *COQ10*, *MVP1*, *RKM3,* and *PRR1*.

## Discussion

Compared with other organisms, the tolerance of yeast to ammonium is unimaginable, and the discovery of ammonium toxicity in yeast occurred significantly later than that of other stresses. It was not until the discovery of the relationship between NH_4_^+^ and K^+^ that the major mystery of how yeast could accomplish this was solved [15]. However, more mechanisms, especially in high concentrations of ammonium under normal K^+^ culture conditions, have not been reported. We aimed to learn more about the mechanisms, through (1) screening of yeast deletion mutant libraries, to identify resistance genes, and (2) transcriptome sequence analysis, to identify transcriptional changes, under high-concentration ammonium treatment. In our results, we found many shared genes and pathways that were involved in resistance to multiple stresses, and also found many genes and pathways that were specific to ammonium resistance.

### Carbon and nitrogen metabolism maintains cellular ammonium resistance

One of the ways in which cells respond to excess intracellular ammonium is to synthesize ammonium into amino acids and exclude the excess to the extracellular space [15, 16]. This process consumes a large amount of carbon skeleton, which is needed for processes such as glycolysis and the TCA cycle. This can be confirmed in the enrichment results of upregulated genes in our transcriptome analysis. Studies by others have come to similar conclusions. For example, In a genome-wide high-throughput screening study of multiple N-nitrosamine compounds using a library of yeast deletion mutants, Joseph Uche Ogbede et al. found that ammonium sulfate caused growth defects in mutants of the arginine biosynthesis pathway [26]. Xueping Tian et al. discovered overall upregulation of starch synthesis and degradation pathways, as well as glycolysis and the TCA cycle, which could provide abundant carbon skeleton material for excess NH_4_^+^ assimilation and avoid carbon deficiency, a mechanism that might also be an important feature of the duckweed response to NH_4_^+^ toxicity [27]. The TCA cycle serves as a mitochondria-based hub for the final steps in carbon skeleton oxidative catabolism for carbohydrates, amino acids, and fatty acids [28]. NH_4_^+^ can be effectively detoxified into amino acids to maintain the availability of the carbon skeleton by utilizing the TCA cycle [29]. Chandran et al. discovered that upregulated genes are mainly enriched in metabolic processes for diverse amino acids as well as nitrogen compounds, by exploring the genomic responses in rice roots dealing with 0.5 mM (NH_4_)_2_SO_4_ [30]. Rui Wang et al. found that a complex physiological and genetic regulatory network of processes including nitrogen metabolism, carbon metabolism, abiotic stress response, and secondary metabolism at the root and leaf levels was involved in NH_4_^+^ resistance in *Myriophyllum aquaticum* [31]. The same response has been observed in mudskippers under NH_4_^+^ stress. Xinxin You et al. discovered that reducing the catabolism of protein and amino acids could be an effective way to slow down internal ammonia accumulation [32]. It is reasonable that genes in various other cellular metabolic processes involving nitrogen could be downregulated. Similar to their results, our upregulated genes were also enriched in these pathways, which indicates that it is possible to improve tolerance to NH_4_^+^ toxicity in yeast by increasing the expression of the TCA cycle, glycolysis, starch and sucrose metabolism (Fig. 5B).

In the GO enrichment analysis of the 34 genes corresponding to sensitive deletion mutants, *URE2*, *GTR1,* and *GTR2* were enriched in several pathways at the highest enrichment level. According to our experimental results, *ure2Δ* is one of two mutants showing significant growth inhibition at 100 mM NH_4_Cl, and *URE2* is not a multidrug resistant gene. The growth of both *gtr1Δ* and *gtr2Δ* was reduced by more than 40% compared with the wild type in the screening results. Ure2 is involved in the inhibition of nitrogen catabolism [33]. When an optimal nitrogen source is available, Ure2 acts as a transcriptional corepressor and downregulates the expression of many genes involved in nitrogen utilization. The Gtr1-Gtr2 GTPase complex is composed of Gtr1 and Gtr2, and is important for sensing the presence of amino acids in the medium by activating TORC1 [34]. Gtr1 is a subunit of the EGO complex, which is responsible for activating TORC1 in response to the utilization of amino acids. The combination of EGO complex and TOR can positively regulate microautophagy [35]. In contrast, *gtr1Δ* reduces the activity of TORC1, which increases the expression level of nitrogen transporters and ammonium consumption but reduces amino acid consumption [36]. *GTR2* encodes a Ras-like small GTPase that plays a role in regulating nutrition-responsive TORC1 kinase signal transduction, exocytosis sorting of endosomes, and epigenetic control of gene expression [37]. Deletion of *GTR1* or *GTR2* may render cells incapable of activating TORC1, resulting in cell toxicity due to the inability to degrade ammonia in vivo under high ammonia stress.

### Osmotic stress is one of the causes of ammonium toxicity

*TPS1* is one of the few genes in our screen for which deletion affects cell growth, but which is significantly upregulated in the transcriptome. *TPS1* encodes trehalose 6-phosphate synthase and catalyzes the first step in trehalose biosynthesis. In *Saccharomyces cerevisiae*, trehalose is a major reserve carbohydrate involved in responses to thermal, osmotic, oxidative, and ethanol stresses [38]. Genes in the trehalose metabolic pathway, including *TPS1*, *TPS2,* and *NTH1,* were all upregulated. *TPS2* is responsible for reaction catalysis of step 2 in trehalose synthesis. The *NTH1* gene product might contribute to trehalose mobilization in *S. cerevisiae* under saline stress conditions [39]. *NTH1,* as well as trehalose biosynthesis genes, was upregulated under saline stress conditions [40]. Cells may be able to reduce the toxicity caused by NH_4_Cl through saline stress by increasing the expression of these genes (*TPS1*, *TPS2,* and *NTH1*)*.*

In addition, Hog1 is an important regulator of transcription in conditions of osmotic stress in yeast [41]. Fang Li et al. found that Hog1 and Slt2 were downregulated under osmotic and cell wall stresses, respectively [42]. Hog1-mediated transcriptional control of *ERG* genes accounts for most of the downregulation of sterol levels upon osmotic stress [43]. In our transcriptome results, the expression of *HOG1*, *SLT2*, *ERG2*, and *ERG6* were all downregulated, and *erg2Δ* and *erg6Δ* were also more sensitive than other mutants, showing severe growth inhibition at 300 mM NH_4_Cl. These results suggest that NH_4_Cl may also be toxic to cells through osmotic stress.

### Mitophagy plays an important role in ammonium resistance

Among the 34 sensitive mutants, KEGG and functional classification were all enriched in the mitophagy pathway, including *yme1Δ*, *slt2Δ,* and *hog1Δ*, of which *yme1Δ* showed severe growth inhibition at 100 mM NH_4_Cl, and *slt2Δ* was significantly inhibited at 300 mM NH_4_Cl. The mitochondrial protease Yme1 plays an important role in the ability of cells lacking tafazzin function to maintain mitochondrial structural integrity, mitochondrial quality control, and mitochondrial autophagy [44]. Yme1 is an ATP-dependent protease located on the inner mitochondrial membrane that is required for the growth of yeast lacking a complete mitochondrial genome [45]. The processing of Atg32 by Yme1 is an important regulatory mechanism of mitophagy [46]. Slt2 and Hog1 are both required for mitophagy. Slt2 is a MAPK of the cell wall integrity pathway and is necessary for the degradation of mitochondria. Hog1, another member of the MAPK family, is a kinase that regulates and is regulated by Sch9p and is independent of the PKA and TOR pathways in response to stress [47]. Hog1 plays a role in Atg32 phosphorylation, and this is necessary for mitophagy [48, 49].

Mitochondria are important organelles that provide cellular energy and the carbon skeleton building blocks for the synthesis of macromolecular substances. Therefore, the removal of damaged mitochondria through mitophagy is essential for maintaining proper cell function [50]. When cells are exposed to ammonium, higher demands are placed on mitochondrial function. This is because, first, every time NH_4_^+^ enters the cell, H^+^ is transported outside the cell, resulting in an increase in intracellular pH [51]. Ammonium uptake requires the consumption of large amounts of ATP for maintaining intracellular pH by using the plasma membrane bound H^+^-ATPase [17].. In addition, studies have found that ineffective transmembrane ammonium cycling in some species can also lead to energy consumption, because a large fraction of intracellular ammonium leaks out of the cell through the membrane. This ineffective transport causes great energy loss, resulting in adverse symptoms [52, 53]. Second, the assimilation of ammonium into amino acids requires a large amount of carbon skeleton, which leads to harmful effects due to insufficient carbon building blocks in the cell [52]. The high concentration of ammonium increases the synthesis rate of glutamic acid, alanine, and glycine, and accelerates the consumption of glucose and glutamine. The increase in the concentration of ammonium salt not only increases the energy requirements of the cells, but also affects the TCA cycle. These all place high demands on mitochondrial function, and the impairment of mitophagy leads to the sensitivity of cells to ammonium toxicity.

## Conclusions

In this study, we analyzed the resistance mechanism that allow yeast to grow under high concentration of ammonium using yeast genome-wide screening. Our results showed that, out of nearly 4200 mutants, 34 mutants were identified and confirmed by drop test as being vulnerable to NH_4_Cl. Furthermore, functional enrichment analysis indicated that these 34 genes were mainly involved in lipid metabolism and ammonia metabolism, post-translational modification, mitophagy, Gtr1-Gtr2 GTPase complex, DNA and RNA synthesis-related genes, cell resistance, and endocytosis-related genes. Transcriptome analysis further supported the accuracy of our screening results and demonstrated that cells significantly upregulated carbon and nitrogen metabolism, TCA cycle and other stress adaptation pathways, and downregulated cell growth-related pathways under NH_4_Cl stress. These results can provide us with a clearer understanding of the resistance mechanism that allow yeast to grow under NH_4_Cl.

## Materials and methods

### Genome-wide screen to identify gene deletion mutants sensitive to NH_4_Cl

The gene deletion library (SGA-V2) was kindly provided by Prof. Charlie Boone, University of Toronto, Canada [54, 55]. The library of non-essential haploid deletion strains containing about 4200 mutants was started from *S. cerevisiae* strain BY4741 (*MATa his3*Δ*1 leu2*Δ*0 met15*Δ*0 ura3*Δ*0*). Each mutant was constructed by replacing the corresponding ORF with a KanMX cassette, and the strains in the library were arranged in 384 format. Mutant *his3Δ*::KanR in this library was designated as the control strain and added as a border around four edges of each plate [56]. The pinning steps for library handling were performed using a SINGER ROTOR HDA Robot (Singer Instruments, UK). The deletion library was cloned on YPD agar plates (with G418 added) and grown at 30 °C for 2 days. The 384 strains in each plate were then transferred to a new agar plate, and each colony was repeated four times to finally form an array of 1536 colonies. The entire library in 1536 array was then cloned onto a solid plate with or without 800 mM NH_4_Cl and grown at 30 °C for 2 days. The growth status of the colonies was photographed by PhenoBooth (Singer Instruments, UK). Images were analyzed using SGAtools (http://sgatools.ccbr.utoronto.ca/) to evaluate the growth of the colonies [57], and compare the growth of each mutant with or without NH_4_Cl. First, images of plates with colonies were processed to give quantified colony sizes for the screen. Next, the colony sizes were normalized and filtered within plates, taking into account position effects and other confounding factors. Ratios of normalized colony sizes from NH_4_Cl-treated and untreated mutants were used as a measure of sensitivity. A score > 0 represented positive interaction, i.e., increased colony size, while a score < 0 represented negative interaction, i.e., decreased colony size. According to previous studies [58], a score of < − 0.2 generally indicates a strong effect, so we used scores < − 0.2 and *P* < 0.05 as thresholds to identify significantly affected mutants. Each experiment was repeated three times.

### Culture conditions

Yeast strains were grown in YPD + G418 medium which contained 1% yeast extract, 2% peptone, 2% glucose, and 200 mg/mL G418. The 800 mM NH_4_Cl medium consisted of 1% yeast extract, 2% peptone, 2% glucose, 200 mg/mL G418, and 800 mM NH_4_Cl. All the strains which were mentioned in this article were cultivated in an incubator at 30 °C.

### Spot assays

For the drop test, we picked up the selected mutant from the SGA-V2 library and cultured it overnight in 3 mL YPD + G418 medium at 30 °C. Then, the strains were diluted to OD_600_ = 0.1, and cultured at 30 °C until the mid-logarithmic stage. The culture was then continuously diluted tenfold in sterile water in 96-well plates and the dilutions were spotted on plates with or without NH_4_Cl. Images were taken after culturing at 30 °C for 2 days, and the growth of yeast colonies was observed. Shown are representative drop tests from three independent replicate assays.

### Bioinformatics enrichment analysis and functional annotation

The GO Term Finder in the Saccharomyces Genome Database (https://www.yeastgenome.org/) was used to analyze the enrichment of GO terms in 34 genes [59]. Then, we used the gene-list analysis tool Metascape (http://metascape.org) with the background of genes corresponding to SGA-V2 library, to conduct KEGG pathway analysis, with a chosen *P*-value of less than 0.05 [60]. The functional annotation results were mapped and clustered using Cytoscape (version 3.8.0) [61].

### Transcriptional RNA sequence analysis

Yeast cells treated with 0 mM and 800 mM NH_4_Cl for 8 hours were used for transcriptome sequencing, and four sample replicates were set for each treatment. Total RNA was extracted using Trizol reagent (Thermofisher, 15,596,018), using the Bioanalyzer 2100 and RNA 6000 Nano LabChip Kits (Agilent, CA, USA, 5067–1511) to analyze the total RNA quantity and purity, the sequencing cDNA library was constructed using high-quality RNA with an RNA integrity number (RIN) > 7.0. The library was sequenced using the Illumina Novaseq™ 6000 sequence platform. Cutadapt (https://cutadapt.readthedocs.io/en/stable/, version: cutadapt-1.9) was used to remove the Illumina adapter contamination and for trimming the reads and clipping the low-quality bases to get high-quality clean reads. Then, sequence quality was verified using FastQC (http://www.bioinformatics.babraham.ac.uk/projects/fastqc/, version: 0.11.9). After that, approximately 6 Gb of clean reads were produced [62]. Differentially expressed genes (DEGs) analysis was performed by DESeq2 software, between two different groups. The genes with the parameter of false discovery rate (FDR) below 0.05 and absolute fold change ≥1.5 were considered differentially expressed genes [63]. Then ClueGO in Cytoscape was used to perform the enrichment analysis of GO function and KEGG pathway for the significantly upregulated genes and the significantly downregulated genes.

## Supplementary Information

**Additional file 1:  Figure. S1.** Drop test of *his∆* in plates with a gradient of concentrations of NH_4_C1.

**Additional file 2: Table S1.** Data from three independent genome-wide screening experiments.

**Additional file 3: Table S2.** GO and KEGG-enriched gene list for the 34 NH4Cl-sensitive mutants.

**Additional file 4: Figure. S2.** Growth curve of BY4741 at different concentrations of NH_4_C1 treatment.

**Additional file 5: Table S3.** Transcriptome sequencing data under 800 mM vs. 0 mM NH_4_Cl treatment.

## Data Availability

All the sequencing data generated in this study have been deposited in the Sequence Read Archive (SRA) database under accession number PRJNA795291 (https://dataview.ncbi.nlm.nih.gov/object/PRJNA795291?reviewer=3v8dgbe27ar7v1vbh4uscmhmvv).
