# Supplementary figures and images for: Genome-wide identification of resistance genes and transcriptome regulation in yeast to accommodate ammonium toxicity
Source: BMC Genomics. 2022 Jul 15;23:514. doi: 10.1186/s12864-022-08742-y (PMC9287935; doi:10.1186/s12864-022-08742-y)

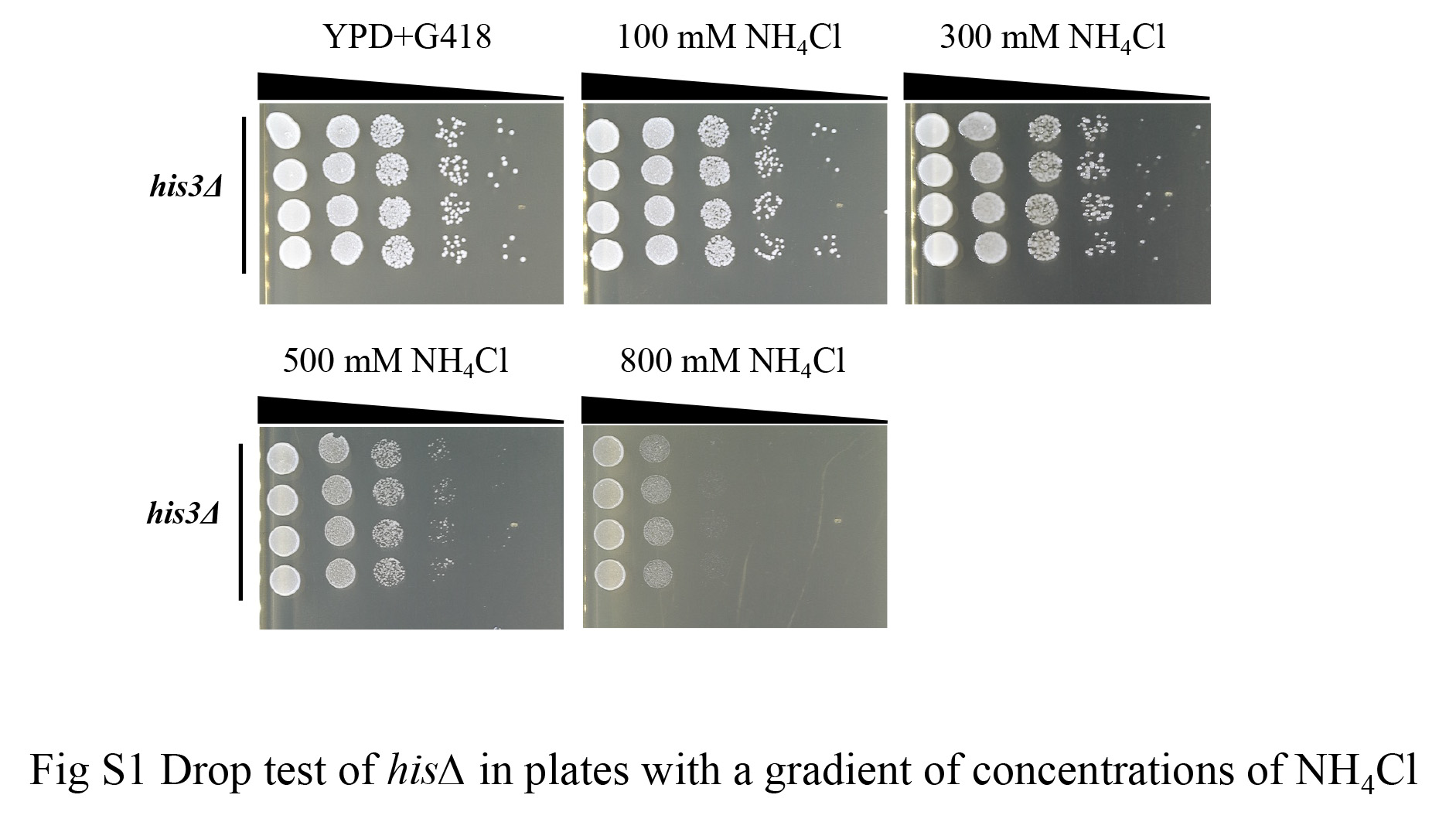

Supplement: Supplementary file 1 — Additional file 1: Figure. S1. Drop test of his∆ in plates with a gradient of concentrations of NH4C1. [file 12864_2022_8742_MOESM1_ESM.jpg]

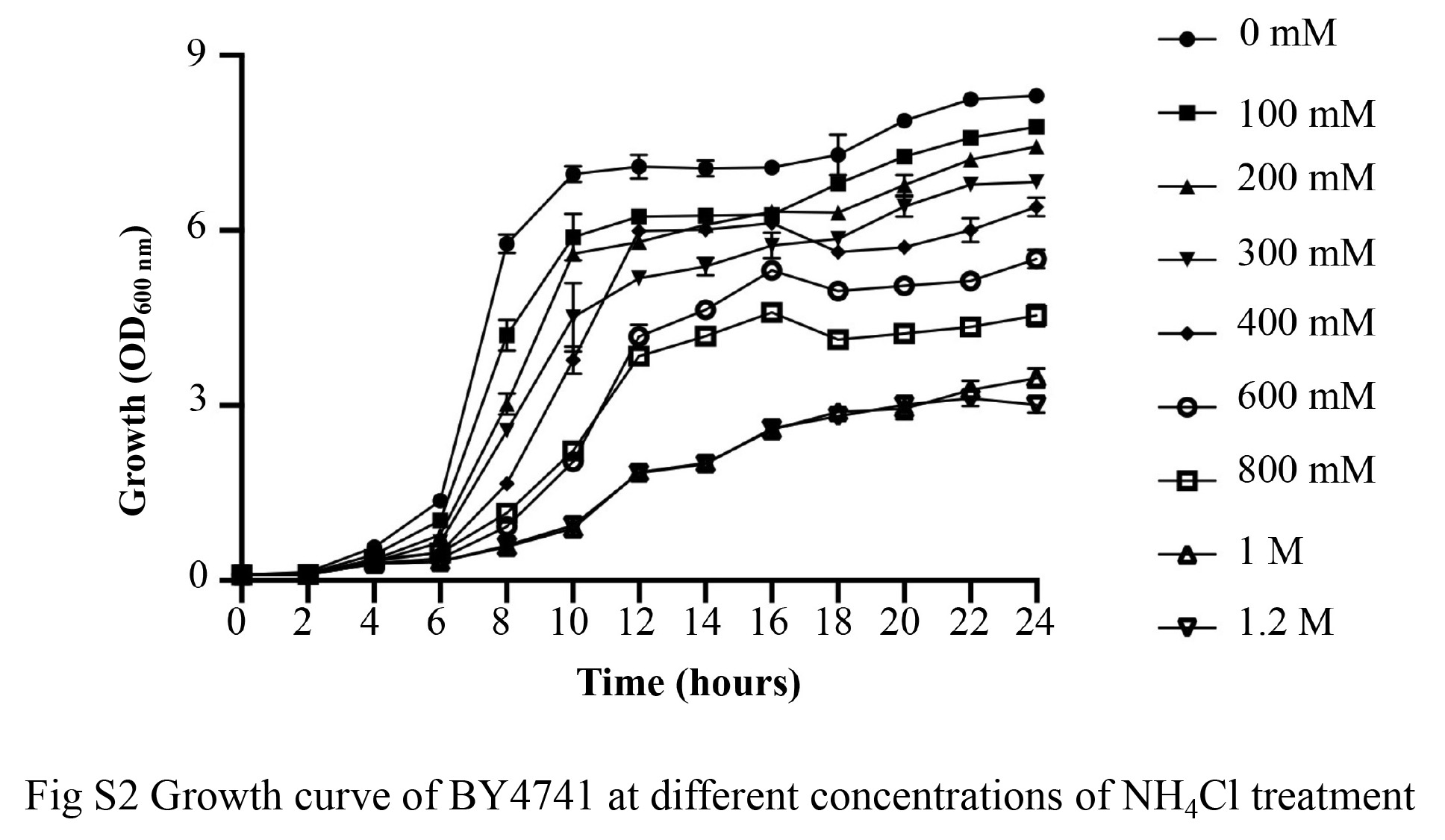

Supplement: Supplementary file 4 — Additional file 4: Figure. S2. Growth curve of BY4741 at different concentrations of NH4C1 treatment. [file 12864_2022_8742_MOESM4_ESM.jpg]
